# Increased Durability of Concrete Made with Fine Recycled Concrete Aggregates Using Superplasticizers

**DOI:** 10.3390/ma9020098

**Published:** 2016-02-08

**Authors:** Francisco Cartuxo, Jorge de Brito, Luis Evangelista, José Ramón Jiménez, Enrique F. Ledesma

**Affiliations:** 1 CERIS/ICIST, DECivil-Instituto Superior Técnico (IST), University of Lisbon, Av. Rovisco Pais, Lisbon 1049-001, Portugal; francisco.cartuxo@gmail.com (F.C.); jb@civil.ist.utl.pt (J.B.); 2 CERIS/ICIST, ADEC-ISEL, Lisbon’s Polytechnic Institute, R. Conselheiro Emídio Navarro, 1, Lisbon 1950-062, Portugal; evangelista@dec.isel.ipl.pt; 3 Construction Engineering Area, EPS de BELMEZ, University of Córdoba, Belmez CP 14240, Spain; efledesma@uco.es; 4 Construction Engineering Area, University of Córdoba, Ed, Leonardo Da Vinci, Campus de Rabanales, Ctra. N-IV, km-396, Córdoba CP 14014, Spain

**Keywords:** fine recycled aggregate, superplasticizers, durability, water absorption, chloride resistance, carbonation resistance

## Abstract

This paper evaluates the influence of two superplasticizers (SP) on the durability properties of concrete made with fine recycled concrete aggregate (FRCA). For this purpose, three families of concrete were tested: concrete without SP, concrete made with a regular superplasticizer and concrete made with a high-performance superplasticizer. Five volumetric replacement ratios of natural sand by FRCA were tested: 0%, 10%, 30%, 50% and 100%. Two natural gravels were used as coarse aggregates. All mixes had the same particle size distribution, cement content and amount of superplasticizer. The w/c ratio was calibrated to obtain similar slump. The results showed that the incorporation of FRCA increased the water absorption by immersion, the water absorption by capillary action, the carbonation depth and the chloride migration coefficient, while the use of superplasticizers highly improved these properties. The incorporation of FRCA jeopardized the SP’s effectiveness. This research demonstrated that, from a durability point of view, the simultaneous incorporation of FRCA and high-performance SP is a viable sustainable solution for structural concrete production.

## 1. Introduction

Concrete is one of the most commonly used materials in the construction sector. Its use has a great environmental impact due to the large amount of natural aggregates and cement required for manufacture. The production of one ton of Portland cement emits between 0.6 and 1.0 tons of CO_2_ [1] which is an emission of 0.2–0.4 tons of CO_2_ per cubic meter of manufactured concrete [2]. Furthermore, consumed aggregates are non-renewable natural resources extracted directly from alluvial deposits or quarries. Their manufacture, handling and transportation also require high energy consumption and emissions.

Concrete structures are demolished at the end of their service life, generating what is known as construction and demolition waste. Using deconstruction techniques [3], very pure concrete waste with a high potential for recycling can be obtained. Concrete wastes from selective demolition are treated in recycling plants, where the steel reinforcement is separated from concrete. The concrete rubble is crushed in an impact or a jaw crusher to reduce the grain size to produce recycled concrete aggregates (RCA). The RCA can be screened to obtain two fractions: coarse recycled concrete aggregates (CRCA) and fine recycled concrete aggregates (FRCA). In the last two decades, numerous studies have analysed the physico-mechanical and chemical properties of RCA and demonstrated their feasibility in the manufacture of new concrete [4].

Most of the research conducted has been focused on the use of CRCA in the manufacture of new structural concrete [5,6,7,8,9,10]. The use of FRCA has been less investigated than that of CRCA in concrete production because of the inferiorphysico-mechanical and chemical properties of this fraction, such as greater amount of cement paste, porosity, water absorption and sulphur compounds [11]. FRCA increases water absorption and chloride penetration in hardened concrete. Carbonation resistance decreased with the incorporation of FRCA [12]. The use of superplasticizers improves the mechanical properties of concrete made with CRCA [13] and FRCA [14], although its effect on the durability of concrete has not been extensively investigated.

For the reasons given above, many international standards allow replacing natural gravel with CRCA but do not allow replacing natural sand with FRCA for structural concrete production [15]. This situation fosters the accumulation of FRCA in landfills and the use of natural sand from river banks or crushed natural rock from quarries, with consequent energy consumption and CO_2_ emissions required from crushing rock at particle size below 4 mm.

Evangelista and de Brito [16], after an extensive review of numerous studies, concluded that, considering the particularities of FRCA in the method used for the mix design and production, it is possible to replace natural sand by FRCA to make structural concrete. However, these authors note that studies concerning the mixing process, constitutive relations for FRCA-concrete and the relationship between different mechanical properties and durability parameters are necessary for the use of FRCA at an industrial scale in the manufacture of structural concrete.

The aim of this study was to experimentally determine the influence of regular superplasticizer and high-performance superplasticizer on the durability properties of concrete made with different replacement ratios of natural sand with FRCA and propose maximum FRCA replacement ratios for each of the superplasticizers tested. No target durability performance was set for the mixes with FRCA. CRCA were not used in order to separate the influence of the FRCA on the results from that of the CRCA.

The importance of this study is that it demonstrates that the use of admixtures significantly improves the durability properties of concrete made with FRCA, whereby the amount of FRCA can be increased. This has the following environmental advantages: (a) the amount of sand collected from rivers and seashores can be reduced; (b) the landfill or illegal deposits of waste concrete can be minimized; (c) the life cycle of concrete can be increased by having a second use in the manufacture of recycled aggregate concrete.

## 2. Literature Review

The SEM images of FRCA particles show a rough surface with mortar adhered to it and numerous pores and micro-cracks [17] that make these aggregates more porous than FNA (fine natural aggregate). This gives two of the most characteristic physical properties of these recycled aggregates: their lower density and higher water absorption. After reviewing fourteen research papers, Evangelista and de Brito [16] showed average values of 2.18 g/cm^3^, 2.28 g/cm^3^ and 2.56 g/cm^3^ for dry-oven (ρ_rd_), saturated surface dry (ρ_ssd_) and apparent (ρ_a_) particle densities respectively, and average values of 9.9% for water absorption at 24 h. A smaller FRCA particle size results in higher water absorption capacity. Guedes *et al.* [18] also found greater angularity and irregular shape of the FRCA particles.

To compensate for the greater water absorption and internal friction between particles derived from its greater angularity, concrete made with FRCA requires more total water than conventional concrete for mixing. This increase in the total w/c ratio is not necessarily harmful, since excess water can be absorbed by the FRCA. However, the increase of water deriving from the greater friction between particles generates an increase in the effective w/c ratio [14]. Concrete made with FRCA shows high porosity and worse microstructure, especially the ITZ between the new cement paste and FRCA [17]. This explains why most of the studies reviewed by Evangelista and de Brito [16] showed that the total or partial replacement of FNA with FRCA reduces the mechanical strengths, increases shrinkage and has a negative effect on the durability of recycled concrete.

The amount of mixing water can be reduced using superplasticizers. The lower w/c ratio improves the compacity of concrete and its mechanical properties [14,19]. However, few studies on durability of concrete made with FRCA and superplasticizers have been carried out. The main problems concerning structural concrete durability are carbonation and the penetration of chloride ions. Aggressive agents (carbon dioxide and chloride ions) move through the capillary pores of the concrete by diffusion, destroying the passive layer and enhancing steel corrosion. There are four main properties of concrete related to its durability: water absorption by immersion, water absorption through capillarity, carbonation resistance and chloride ion penetration. The main studies carried out on the effect of FRCA on the durability of concrete are described below.

Evangelista and de Brito [11,12], using a reference concrete (RC) of 60 MPa made with CEM-I 42.5 R, replaced in volume 30% and 100% of FNA with FRCA. A modified carboxilate based superplasticizer and w/c ratios of 0.41 and 0.48 were required for reference concrete and recycled concrete to keep a constant slump of 80 ± 10 mm. Compressive strength at 28 days decreased 3.7% and 7.6% for each replacement ratio; water absorption by immersion increased by 16.8% and 46% respectively. Capillary water absorption was more affected by the use of FRCA than water absorption by immersion. Sorptivity increased 34.4% and 70.3% in concrete made with 30% and 100% FRCA respectively. The non-steady-state migration coefficient of chlorides (D_nssm_) grows linearly when replacement ratio increases, obtaining values ranging between 17.99 × 10^−12^ m^2^/s for RC and 24.07 × 10^−12^ m^2^/s for 100% FRCA-concrete. The carbonation depth showed a similar trend to chloride penetration. Increases of 40% and 110% were observed for 30% and 100% replacement ratio respectively.

Kou and Poon [20] in a first study replaced FNA with FRCA in weight. Five levels were used: 0%, 25%, 50%, 75% and 100%. The design method considered the aggregates’ saturated surface dry. No superplasticizers were used and water was added during the mix. The authors found in a first series that at a constant w/c = 0.53 the slump increased with the increase of FRCA content. This was justified by the greater amount of free water not absorbed by the aggregates during or after mixing, which increased the fluidity of the fresh concrete. Replacement ratios of 100% decreased the compressive strength by 24% at 28 days. In a second series water was added to obtain a fixed slump of 60–80 mm. In this case the compressive strength decreased 29% for the 100% replacement level. In both series the resistance to chloride-ion penetration decreased as the FRCA content increased. All mixes showed better resistance at 90 days than 28 days.

In a second study, Kou and Poon [21] replaced in volume FNA with fine recycled aggregates (FRA). The same replacement ratios were tested. In a first series, CEM-I and a superplasticizer and a viscosity agent were used to make self-compacting concrete (SCC). The flow diameter increased as the fine recycled aggregate content increased, because more water was added into the mixtures initially to compensate for the higher water absorption of FRCA. The slump loss increased with increasing FRA content, due to the higher water absorption capacity of FRA which took up the free water quickly. The fresh concrete density decreased as the FRA content increased. The authors showed that the incorporation of 25% and 50% of FRA did not significantly affect the 28-day compressive strength of SCC mixes while replacement ratios of 100% decreased the compressive strength by 10%. Contrary to compressive strength, the resistance to chloride ion penetration increased with FRA content. This was justified by the filler effect of FRA. The resistance to chloride ion penetration increased as the w/c decreased.

Zega and Maio [22] studied the durable behaviour of structural concrete made with three replacement ratios of FNA with FRCA: 0%, 20% and 30%. A water-reducing admixture was used at a variable rate to keep the (w/c) constant at 0.45. Concrete made with 30% replacement ratio showed a decrease of 65% for slump and 5% for compressive strength. The water absorption by immersion increased 15%, although sorptivity showed similar values to those of the reference concrete. The carbonation depths of specimens located in an industrial environment were similar to those of conventional concrete after 620 days. Very low values were obtained, which was attributed to the medium level of aggressiveness of the natural environment. This justifies the need for accelerated tests to detect differences.

Sim and Park [23] made a reference concrete of 40 MPa with w/c = 0.35. Six replacement levels of FNA with FRCA were tested: 0%, 30%, 50%, 70% and 100%, in concrete made with coarse RCA. A polycarbonate based superplasticizer was used. At 100% of replacement level the compressive strength decreased by about 33%. These authors found that the chloride ion penetration was not affected by the use of FRCA. No clear conclusions were obtained on the effect of FRCA on carbonation.

Pereira *et al.* [14,19] conducted one of the first studies on the effect of a regular superplasticizer and a high-performance superplasticizer on the workability and mechanical performance of concrete made with FRCA. Based on the Faury method five replacement ratios in volume were tested: 0%, 10%, 30%, 50% and 100%. These authors demonstrated the efficiency of superplasticizer in terms of workability and mechanical performance when FRCA were used. Durability studies were not included.

Geng and Sun [17] evaluated the effect of the particle size and the FRCA amount on the carbonation resistance of concrete made with four replacement ratios: 0%, 20%, 40% and 80%. Two series were tested. In the first one, a constant w/c ratio (0.4) was used. With this ratio a poor workability was obtained, which increases the micro-flaws. In a second series a poly-carboxylic acid water reducer and variable amount of water was used to obtain a slump of 180 ± 10 mm. In both series, the carbonation depth increased with the amount of FRCA because of the higher porosity of recycled concrete. Concrete made with smaller particle size showed lower carbonation resistance because of the higher amount of old cement paste.

## 3. Experimental Program

Two commercially available superplasticizers widely used in Portugal were selected to study the influence of superplasticizers on the durability of concrete made with FRCA: Sikament 400 plus (SP1) and SikaPlast 898 (SP2).

SP1 is a regular superplasticizer chemically based on organic polymers and admixtures that works by electrostatic repulsion. SP2 is a high-performance superplasticizer chemically based on a combination of modified polycarboxylates in an aqueous solution that works by electrostatic, and basically, by steric repulsions. Their density (23 ± 2 °C) is 1.22 ± 0.02 kg/dm^3^ and 1.07 ± 0.02 kg/dm^3^ respectively, and their pH (23 ± 2 °C) is 9.0 ± 1.0 and 5.0 ± 1.0, respectively.

Three families of concrete were made:

- C0: concrete made without SP;
- C1: concrete made with SP1;
- C2: concrete made with SP2.

Five replacement ratios of fine natural aggregate (FNA) with FRCA were tested for each of the three families of concrete (Ci): 0%, 10%, 30%, 50% and 100%. These percentages are consistent with the replacement ratios used in two previous works by Pereira *et al.* [14,19]. A total of 15 mixes were made. Table 1 shows the nomenclature of the mixes.

**Table 1:** Concrete mixes.

Replacement Ratio	Without Plasticizer	Superplasticizer SP1	High-Performance Superplasticizer SP2
0%	RC0	RC1	RC2
10%	C0.10	C1.10	C2.10
20%	C0.30	C1.30	C2.30
50%	C0.50	C1.50	C2.50
100%	C0.100	C1.100	C2.100

### 3.1. Aggregates Characterization

Two commercial limestone crushed gravels: medium gravel 6/12 mm (CNA1) and coarse gravel 12/20 mm (CNA2), two commercial washed siliceous sands: fine sand 0/2 mm (FNA1) and coarse sand 0/4 mm (FNA2) and one FRCA obtained from crushed concrete blocks were used to manufacture the concrete mixes.

In order to control the original properties of the concrete, a mix of concrete type X0 (P) CL0.40 D_max_ 22 S2 (C30/37 according to NP EN 206-1:2007 [24]) was manufactured by a ready-mixed concrete company (Unibetão S.A., Lisbon, Portugal) under controlled conditions. Its composition and mechanical properties have been described in a previous work [13]: 1931 kg/m^3^ of aggregates, 256 kg/m^3^ of CEM IV/B, w/c ratio of 0.57; the mean strength at 28 days was 41.4 MPa. Concrete blocks were crushed in a laboratory using a jaw crusher after 30 days of curing. The crushed material was sieved though a 4 mm sieve to obtain the 0/4 mm fraction (FRCA).

Table 2 shows the physico-mechanical properties of natural aggregates from a quarry and FRCA. According to the literature, recycled aggregate showed the lowest oven-dry density value, the highest relationship ρ_ssd_/ρ_rd_ value and much higher water absorption.

**Table 2:** Physico-mechanical properties of natural and recycled aggregates.

Characteristic	Units	Standard	FRCA ^1^	FNA-1	FNA-2	CNA-1	CNA-2
D_max_	mm	EN 933-2:1999 [25]	4	1	4	11.2	22.4
Oven-dry particles density	ρ_rd_ (kg/m^3^)	EN 1097-6:2003 [26]	2298	2674	2667	2570	2639
Saturated surface-dry particles density	ρ_ssd_ (kg/m^3^)	EN 1097-6:2003 [26]	2460	2678	2674	2600	2665
ρ_ssd_/ρ_rd_	-	-	1.07	1.00	1.00	1.01	1.01
Loose bulk density	(kg/m^3^)	EN 1097-6:2003 [26]	1393	1583	1542	1362	1370
Voids content	%	EN 1097-6:2003 [26]	39.4	40.8	42.2	47	48.1
Water absorption	WA_24_ (%)	EN 1097-6:2003 [26]	7.09	0.15	0.26	1.17	0.98
Shape index	%	EN 933-4:2002 [27]	-	-	-	17.4	15.7
Los Angeles coefficient	%	EN 1097-2:2002 [28]	-	-	-	27.2	25.6
Fineness modulus	-	EN 933-1:2000 [29]	-	2.01	3.87	6.63	7.29

^1^ Fraction 0/4 mm.

**Figure 1:**
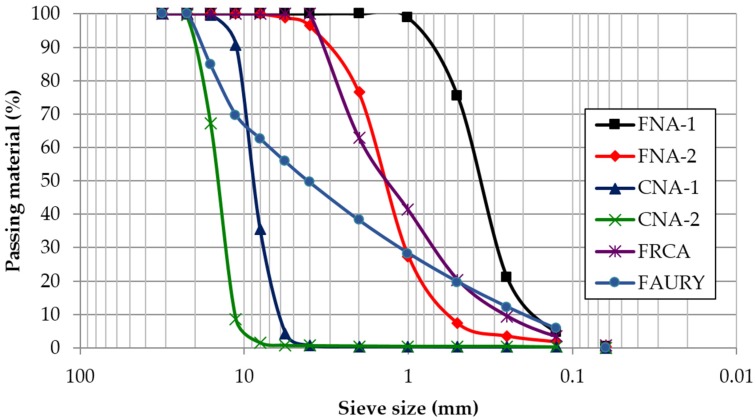
Particle size distribution of the natural aggregate (NA), and fine recycled concrete aggregate (FRCA) and FAURY curve.

Commercial natural aggregates showed more homogeneous grading curves than FRCA (Figure 1). Continuous grading curves are a typical property of recycled aggregates from CDW that allows high compacity [30,31], hence, FRCA had the lowest percentage of voids in the bulk density test (Table 2).

### 3.2. Design Criteria and Production of the Mixes

The mixes were designed according to the Faury method. According to NP EN 206-1:2007 [24] the following data were considered to design the reference concrete (RC0): exposure class XC3, strength class C 25/30, slump class S3 (100 a 150 mm) and CEM-I 42.5 R. The mixes were designed according to the following criteria:

Criterion-1: Adjust the particle size based on Faury’s method. With the aim of obtaining a curve of maximum compacity and eliminating the effect of particle size on the properties of the concrete, the mass of each of the aggregates was calculated beforehand, according to the Faury method. Fine aggregates were sieved and separated in the following particle sizes: 0.063 mm, 0.125 mm, 0.25 mm, 0.5 mm, 1 mm, 2 mm and 4 mm. The amounts for each of these fractions were incorporated into the mix thus achieving a perfect fit to the Faury curve.

Fine natural aggregates were replaced by volume for each of the size fractions according to the following equation:

(1) $$M_{FRCA}=\%i\times M_{FNA}\times \frac{\delta_{FRCA}}{\delta_{FNA}}$$

where:

- M_FRCA_—Mass of FRCA (kg);
- M_FNA_—Mass of FNA (kg);
- %*i*—FRCA replacement ratio (%);
- δ_FRCA_—Oven-dry density FRCA (kg/dm^3^);
- δ_FNA_—Oven-dry density FNA (kg/dm^3^).

Criterion-2: Use a fixed amount of superplasticizer in each of the concrete families. The superplasticizers SP1 and SP2 were added at a fixed proportion of 1% by weight of cement; this value is within the range recommended by the manufacturer of the products. Both admixtures meet the technical specifications of the UNE-EN 934-2:2001 [32]. The admixtures’ properties and the specific requirement according to the European Standard were described in a previous work [13].

Criterion-3: Adjust the water/cement ratio to obtain a similar slump. The amount of water was adjusted to achieve a similar slump (using the Abrams cone) of 125 ± 15 mm in all mixes tested. The higher FRCA water absorption should be compensated in mixes made with this recycled material. For this purpose the FRCA water absorption over time was studied beforehand, following the method proposed by Leite [33]. In the first 10 min of estimated mixing period, the FRCA had absorbed 77.4% of its potential capacity. Table 3 presents the composition of each concrete mix.

**Table 3:** Composition of 1 m^3^ of each concrete mix.

Mixes		RC0	C0.10	C0.30	C0.50	C0.100	RC1	C1.10	C1.30	C1.50	C1.100	RC2	C2.10	C2.30	C2.50	C2.100
Replacement ratio (%)		0	10	30	50	100	0	10	30	50	100	0	10	30	50	100
Cement (kg)		350.0	350.0	350.0	350.0	350.0	350.0	350.0	350.0	350.0	350.0	350.0	350.0	350.0	350.0	350.0
Water (l)		178.5	183.6	186.7	193.6	209.9	150.5	159.1	169.3	179.4	192.9	133.0	138.0	141.5	148.3	160.8
w/c ratio ^1^		0.51	0.52	0.53	0.55	0.60	0.43	0.45	0.48	0.51	0.55	0.38	0.39	0.40	0.42	0.46
(w/c)_ef_ ratio ^2^		0.51	0.52	0.52	0.53	0.55	0.43	0.45	0.47	0.49	0.50	0.38	0.39	0.39	0.40	0.41
FNA (kg)	Total	900.8	806.6	626.5	446.2	0.0	942.6	840.3	646.2	455.8	0.0	969.7	862.9	676.0	480.2	0.0
FRCA (kg)	Total	0.0	77.1	231.1	384.0	757.7	0.0	80.3	238.3	392.2	780.2	0.0	82.9	249.3	413.2	820.4
CNA-1 (kg)		237.0	236.0	236.0	235.0	233.0	246.0	243.0	241.0	239.0	238.0	251.0	248.0	248.0	248.0	247.0
CNA-2 (kg)		690.0	688.0	688.0	684.0	678.0	714.0	709.0	702.0	696.0	694.0	730.0	727.0	727.0	724.0	721.0
Superplasticizer (kg)		0.0	0.0	0.0	0.0	0.0	3.5	3.5	3.5	3.5	3.5	3.5	3.5	3.5	3.5	3.5
Slump (mm)		122.5	126.0	122.0	129.5	125.5	122.0	129.5	125.0	122.5	119.0	123.5	126.0	127.5	126.0	137.0

^1^ w/c ratio: total water in the mix/cement content; ^2^ (w/c) effective ratio: total water in the mix discounting the water absorbed by the FRCA in 10 min.

Criterion-4: No pre-saturated FRCA.

Based on the studies of Ferreira *et al.* [7], Poon *et al.* [34] and Tam *et al.* [35], FRCA were not previously pre-saturated and they were used with their natural moisture (3.2%). The mixing process was done in the following order: FNA and FRCA were introduced into the mixer together with the amount of water absorption of FRCA estimated for a period of 10 min and 2/3 of the mixing water. After 4 min the CNA-1 and CNA-2 were added and mixed for 2 min to homogenize the mix. At minute 6, the cement and the remaining water mixed with the SP were added and mixed for 4 min. In all mixes, the Abrams cone test was performed to ensure that the slump was within the target limits.

### 3.3. Specimen Preparation and Testing Procedures

The evaluation of the fresh concrete properties is fundamental since they have a significant impact on the performance and durability of hardened concrete. Two properties were tested in the fresh state: workability (using the Abram cone) according to NP EN 12350-2:2006 [36] and the specific density according to NP EN 12350-6:2006 [37].

The compressive strength according to UNE EN 12390-3:2009 [38] was measured to characterize the mechanical properties of hardened concrete. For this purpose five cubic specimens of 150 mm × 150 mm × 150 mm were used. The specimens were cured for 28 days in a humidity chamber (chamber-1) programmed to maintain a temperature of 20 ± 2 °C and a relative humidity of 95% ± 5%.

The following durability properties of concrete were studied: water absorption by immersion, water absorption by capillarity, chloride penetration and carbonation resistance.

Water absorption by immersion measures the capacity of a fluid to move through the pore structure of concrete and it is mainly related to the open pore structure. This is one of the most important properties in terms of durability. Water absorption by immersion was tested according to standard LNEC E394:1993 [39] established by the Portuguese National Laboratory of Civil Engineering (LNEC). Four cubic specimens 100 mm × 100 mm × 100 mm were used. The specimens were cured for 28 days in a wet chamber. The testing methodology was described in detail by Evangelista and de Brito [12].

Capillary absorption is a phenomenon by which a fluid moves through the microstructure of concrete induced by the interaction between molecules of the fluid and the surface pressure of the concrete’s capillaries. According to Jurin’s law, capillary movement increases with decreasing capillary diameter. Capillary absorption is related to the diffusion of chloride and carbonation of concrete [40]. This property was tested in accordance with Portuguese standard LNEC E393:1993 [41]. Three cylindrical specimens with a radius of 75 mm and a height of 100 mm were used. The specimens were cured for 28 days in a wet chamber. Prior to testing, the samples were dried for 14 days in a ventilated oven at a temperature of 60 ± 5 °C. The testing methodology is described in detail in Evangelista and de Brito [12].

**Figure 2:**
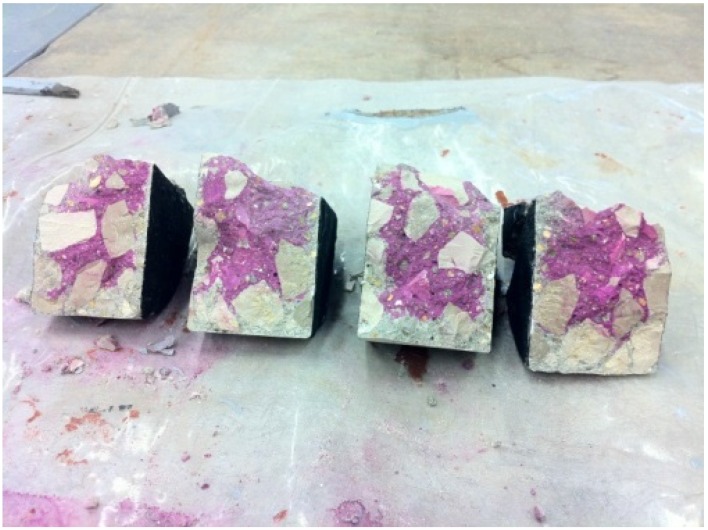
Carbonation resistance test.

Carbonation is a chemical reaction between the calcium hydroxide cement and carbon dioxide from the air. The carbon dioxide reacts with the water in the porous structure to form carbonic acid, which reacts with calcium hydroxide and water to obtain calcium carbonate. Carbonation reduces the pH of the concrete and enhances steel corrosion. Carbonation resistance was tested according to Portuguese standard LNEC E391:1993 [42]. Four cylindrical specimens with a diameter of 100 mm and a height of 40 mm were tested. Samples were obtained from specimens 250 mm high submerged in water for 14 days. After cutting the samples, they remained another 14 days in a dry chamber at 20 ± 2 °C and relative humidity of 50% ± 5%. After 28 days, the circular sections of the specimens were painted with an insulating rubber, so the carbonation was only possible for the lateral side. The specimens were tested at 7, 28, 56 and 91 days after introduction into the carbonation chamber under the following conditions: CO_2_ concentration of 5% ± 0.1%, temperature of 23 ± 3 °C and relative humidity of 50% ± 5%. Each specimen was broken into four parts and immediately sprayed with a phenolphthalein solution. The maximum and mean carbonation depth was measured in each fragment (Figure 2).

**Figure 3:**
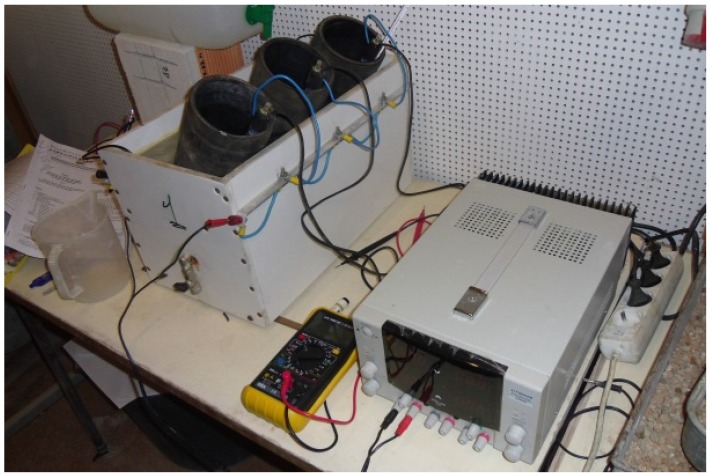
Equipment to measure chloride penetration.

The presence of chloride ion in concrete is related to the corrosion of steel. From a content of chlorides, the passivation layer on the surface of steel in concrete is destroyed and the conditions for the onset of corrosion occur. An accelerated chloride penetration test was carried out in accordance with standard LNEC E463:2004 [43] (Figure 3). Evangelista and de Brito [12] described in details the methodology used and the mathematical expression to determine the non-steady-state migration coefficient of the chlorites (D_nssm_). Three cylindrical specimens with a diameter of 100 mm and a height of 50 mm cut from specimens of 250 mm height and the same diameter were tested. Samples were cured for 14 days in a wet chamber at 20 ± 2 °C and relative humidity of 95% ± 5%. After cutting, the samples were placed in a dry chamber at a temperature of 20 ± 2 °C and relative humidity of 50% ± 5%. The tests were carried out at two ages: 28 and 91 days.

## 4. Results and Discussion

### 4.1. Workability

Table 3 shows the average of the Abrams cone slump results for each of the mixes. Table 4 shows the (w/c) effective and it also gives the percentage of variation due to the use of FRCA for each concrete family (Δ_FRCA_) and the percentage of variation due to the use of superplasticizers SP1 and SP2 with respect to the superplasticizer-free concrete (Δ_SP_).

**Table 4:** Effective w/c ratio of each of the concrete’s families and FRCA replacement ratios.

Family	C0			C1			C2		
FRCA (%)	(w/c) Effective	Δ_FRCA_ (%)	Δ_SP_ (%)	(w/c) Effective	Δ_FRCA_ (%)	Δ_SP_ (%)	(w/c) Effective	Δ_FRCA_ (%)	Δ_SP_ (%)
0	0.51	0.00	0.00	0.43	0.00	−15.69	0.38	0.00	−25.49
10	0.52	1.96	0.00	0.45	4.65	−13.46	0.39	2.63	−25.00
30	0.52	1.96	0.00	0.47	9.30	−9.62	0.39	2.63	−25.00
50	0.53	3.92	0.00	0.49	13.95	−7.55	0.40	5.26	−24.53
100	0.55	7.84	0.00	0.50	16.28	−9.09	0.41	7.89	−25.45

The use of superplasticizers reduced the effective (w/c) ratio for all replacement ratios of FNA with FRCA. The lowest values of (w/c)_ef_ were achieved in concrete made with SP2. The (w/c)_ef_ ratio increased with the use of FRCA for the three concrete families, which is explained by the greater angularity of the recycled particles and the need to use more water to achieve the same slump. Concrete made with SP1 reduced the (w/c)_ef_ ratio with respect to RC0 concrete family between 15.7% and 9.09% for replacement rates of 0% and 100%, respectively, while concrete made with SP2 maintained a reduction in the (w/c)_ef_ ratio of around 25% for every replacement ratio. This shows that SP1 lost effectiveness for increasing percentages of FRCA, while SP2 was impervious to that effect.

Similar results were obtained by Pereira *et al.* [14]. These authors attributed the loss of effectiveness of SP1 to the higher specific surface of cement present in concrete with FRCA, since these lignosulfonate-based superplasticizers act mainly by electrostatic repulsion onto the surface of the cement particles. On the other hand, the dispersion phenomenon in polycarboxylic acids superplasticizers (SP2) is mainly due to the steric hindrance effect, less affected by the surface of cement particles.

### 4.2. Bulk Density of Fresh Concrete

Table 5 shows the average of the bulk density of fresh concrete for each of the mixes and two columns with the effect of FRCA (Δ_FRCA_) and of the superplasticizers (Δ_SP_).

**Table 5:** Fresh concrete density of each of the concrete’s families and FRCA replacement ratios.

Family	C0			C1			C2		
FRCA (%)	Density (kg/m^3^)	Δ_FRCA_ (%)	Δ_SP_ (%)	Density (kg/m^3^)	Δ_FRCA_ (%)	Δ_SP_ (%)	Density (kg/m^3^)	Δ_FRCA_ (%)	Δ_SP_ (%)
0	2372.40	0.00	0.00	2438.80	0.00	2.80	2437.00	0.00	2.72
10	2361.90	−0.44	0.00	2429.90	−0.36	2.88	2431.30	−0.23	2.94
30	2358.50	−0.59	0.00	2417.80	−0.86	2.51	2426.50	−0.43	2.88
50	2318.70	−2.26	0.00	2368.60	−2.88	2.15	2391.20	−1.88	3.12
100	2295.10	−3.26	0.00	2347.40	−3.75	2.28	2346.90	−3.70	2.26

In all concrete families, density decreased when increasing the use of FRCA, which is justified by the lower density of the FRCA [16].

For each replacement ratio, the concrete families made with SP1 and SP2 showed higher density than the superplasticizer-free family (C0). This is justified by the lower (w/c)_ef_ ratio in concrete families C1 and C2, although the differences between C1 and C2 were small.

Most authors have shown that the density of concrete made without admixtures decreases linearly with the rate of recycled aggregates [34,44]. Pereira *et al.* [14] showed second-degree polynomial curves to explain the effect of replacement ratio on the relative density of FRCA-concrete.

### 4.3. Compressive Strength

Table 6 shows the average compressive strength at 28 days (f_cm_) for each of the mixes and the values of Δ_FRCA_ and Δ_SP_ defined above.

For each of the concrete families (CO, C1 and C2), the compressive strength decreased for higher FRCA contents, except for concrete family C0 at 10% replacement ratio, which can be explained by the possible filler effect of the very fine broken particles from FRCA generated during the mixing process that offsets the higher porosity of the recycled aggregates [45]. The compressive strength of the mix made with 100% replacement ratio fell around 17%, 29% and 20% relative to RC0, RC1 and RC2, respectively, which shows the greater sensibility of SP1 to the use of FRCA.

**Table 6:** 28-day compressive strength of each of the concrete’s families and FRCA replacement ratios.

Family	CO			C1			C2		
FRCA (%)	Compressive Strength (MPa)	Δ_FRCA_ (%)	Δ_SP_ (%)	Compressive Strength (MPa)	Δ_FRCA_ (%)	Δ_SP_ (%)	Compressive Strength (MPa)	Δ_FRCA_ (%)	Δ_SP_ (%)
0	49.37	0.00	0.00	66.79	0.00	35.29	80.64	0.00	63.33
10	51.17	3.65	0.00	63.86	−4.39	24.80	77.41	−3.99	51.29
30	47.21	−4.38	0.00	61.65	−7.69	30.60	71.73	−11.04	51.95
50	43.53	−11.83	0.00	58.73	−12.06	34.94	69.31	−14.05	59.23
100	41.20	−16.54	0.00	47.36	−29.10	14.93	64.72	−19.74	57.07

For each replacement ratio, the use of superplasticizers increased compressive strength. This is due to the lower (w/c)_ef_ ratio of the mixes made with SP (Table 4). It is significant that the concrete made with 100% FRCA and SP2 (C2.100) showed an increase in the compressive strength of 31.1% relative to the reference concrete (RC0) made with FNA only. This is justified because the (w/c)_ef_ ratio was 19.6% lower in C2.100 than RC0 (Table 4).

Pereira *et al.* [14] found a lesser effect of FRCA on the compressive strength. These authors state that the total replacement of FNA with FRCA reduced 2.3%, 15.4% and 3.4% the compressive strength at 28 days for concrete made without SP, with SP1 and with SP2, respectively.

### 4.4. Water Absorption by Immersion

**Figure 4:**
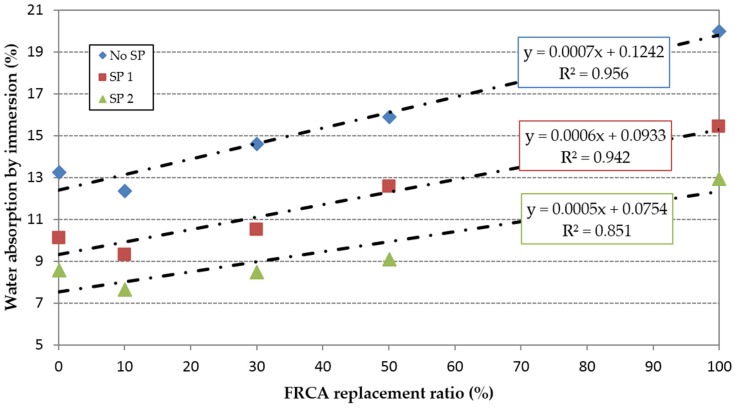
Water absorption by immersion.

Figure 4 shows that water absorption by immersion increased linearly when the replacement ratio of FRCA increased in all concrete families. This is associated with the higher porosity of the mixes made with FRCA. One exception to this trend occurred for the 10% replacement ratio, which resulted in a decrease in the water absorption of 7%, 7.8% and 11.3% relative to the reference concrete mixes RC0, RC1 and RC2 respectively. This can be attributed to a filler effect of the broken particles from the FRCA during the mixing process that offsets the higher porosity of the recycled aggregates. The water absorption decreased between 21% and 28% in the concrete family made with SP1 and between 35% and 43% in the concrete family made with SP2. Concrete made with 100% replacement of FNA with FRCA and SP2 dropped the water absorption by immersion a 2.4% relative to the reference concrete (RC0), while increases of 16.3% were observed with the use of SP1.

**Figure 5:**
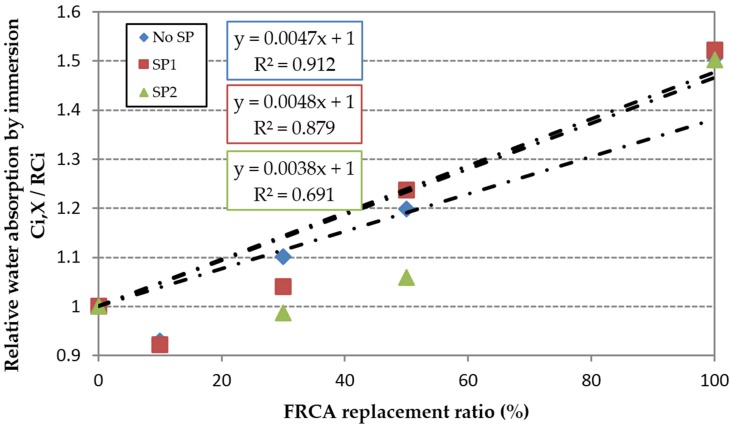
Influence of FRCA replacement ratio on the relative water absorption by immersion.

In relative terms the water absorption by immersion was similarly affected by the use of FRCA in the three families of concrete, since the slopes of the linear trends were very similar (Figure 5); as can be seen, the linear trend when SP was used is not so clear.

**Figure 6:**
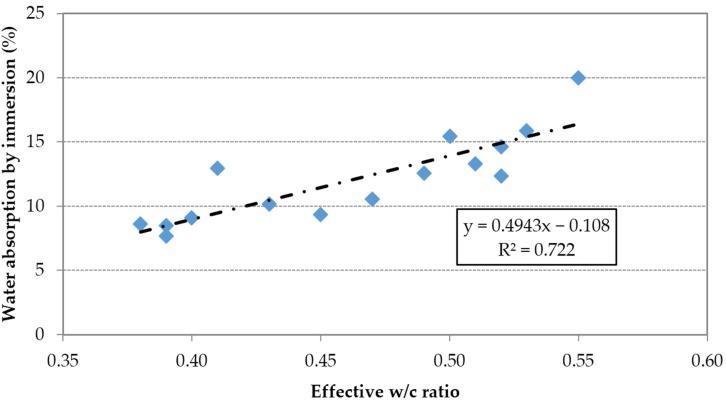
Water absorption by immersion *vs.* effective w/c ratio.

**Figure 7:**
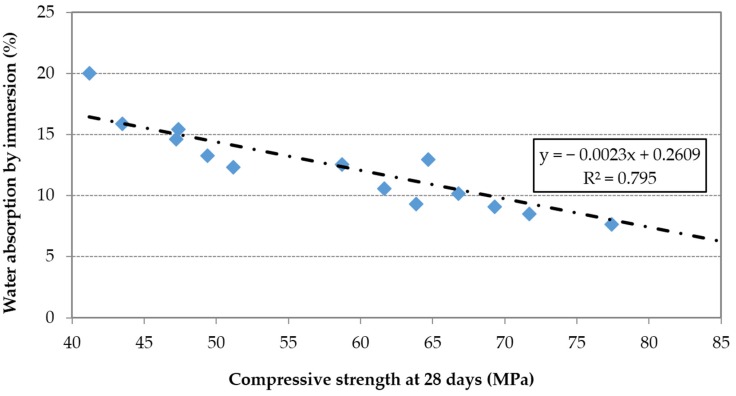
Water absorption by immersion *vs.* 28-day compressive strength.

Using the results of the 15mixes, a direct relationship between water absorption by immersion and effective w/c ratio can be observed (Figure 6). Furthermore, this property was inversely proportional to the compressive strength (Figure 7).

According to the literature review, manufacturing of concrete made with FRCA without admixtures or conventional superplasticizers significantly increased the water absorption by immersion parameter [12,22]. So, in view of the results obtained, it is possible to make structural concrete with FRCA and high-performance superplasticizer maintaining the water absorption by immersion values. This is an unprecedented result in the literature.

### 4.5. Capillarity Water Absorption

**Figure 8:**
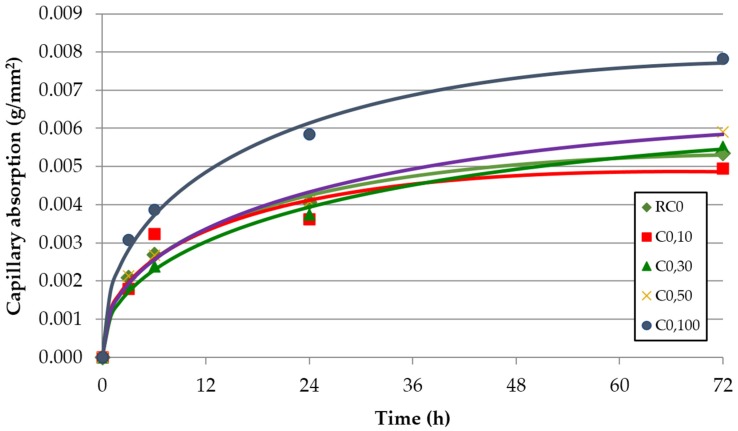
Capillary water absorption over time—concrete family C0 (without SP).

**Figure 9:**
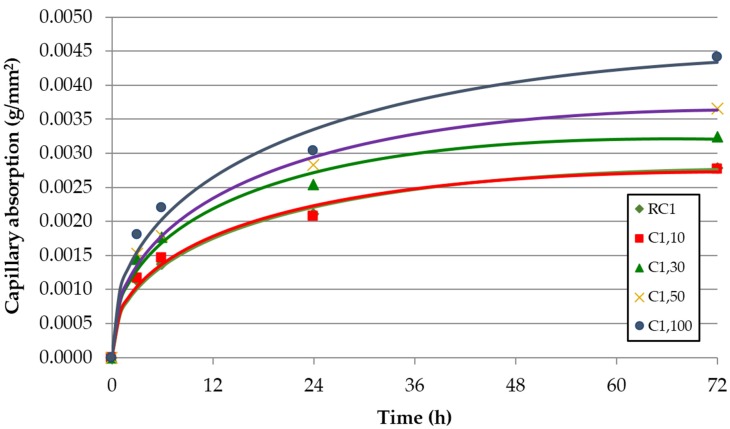
Capillary water absorption over time—concrete family C1 (with SP1).

**Figure 10:**
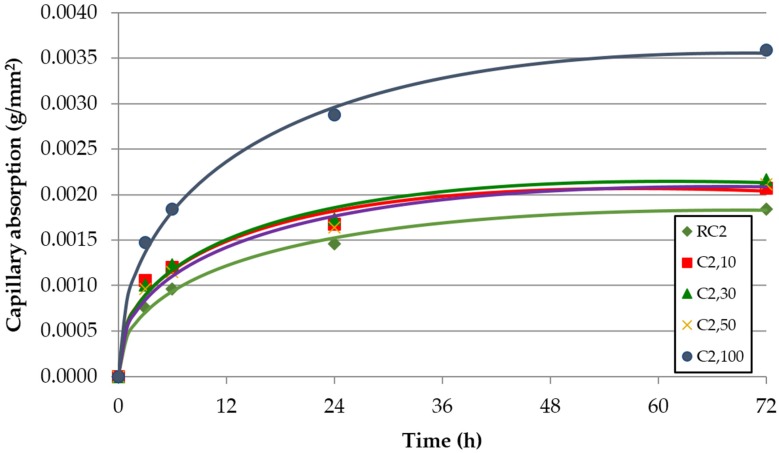
Capillary water absorption over time—concrete family C2 (with SP2).

The evolution over time of the capillarity water absorption for each of the concrete families and replacement ratio of FRCA is shown in Figure 8, Figure 9 and Figure 10. These graphs represent the results from each test and the adjustment function given by Halls [46]:

(2) $$W=A+S\times t^{1/2}-C\times t$$

where W is the capillary water absorption; t is time; S is sorptivity; and A and C are constants. According to Evangelista and de Brito [12], the water absorption by capillarity agrees quite well with the Hall’s model. Table 7 shows the adjustment parameters and sorptivity values.

**Table 7:** Adjustment parameters of the Hall’s capillary model.

Mixes	A	C	S	ΔS (%)	*R*^2^
**RC0**	1.16 × 10^−4^	6.46 × 10^−5^	1.16 × 10^−3^	-	0.995
**C0,10**	1.66 × 10^−4^	7.04 × 10^−5^	1.15 × 10^−3^	−0.9	0.947
**C0,30**	1.33 × 10^−4^	4.15 × 10^−5^	0.98 × 10^−3^	−15.5	0.994
**C0,50**	1.57 × 10^−4^	5.07 × 10^−5^	1.10 × 10^−3^	−5.2	0.992
**C0,100**	1.90 × 10^−4^	9.12 × 10^−5^	1.66 × 10^−3^	43.1	0.994
**RC1**	7.00 × 10^−5^	3.32 × 10^−5^	5.99 × 10^−4^	-	0.993
**C1,10**	9.74 × 10^−5^	3.50 × 10^−5^	6.07 × 10^−4^	1.3	0.986
**C1,30**	1.08 × 10^−4^	4.66 × 10^−5^	7.61 × 10^−4^	27.0	0.988
**C1,50**	9.09 × 10^−5^	4.59 × 10^−5^	8.07 × 10^−4^	34.7	0.993
**C1,100**	1.98 × 10^−4^	4.34 × 10^−5^	8.56 × 10^−4^	42.9	0.976
**RC2**	3.63 × 10^−5^	2.57 × 10^−5^	4.29 × 10^−4^	-	0.996
**C2,10**	9.67 × 10^−5^	3.42 × 10^−5^	5.19 × 10^−4^	21.0	0.976
**C2,30**	7.96 × 10^−5^	3.37 × 10^−5^	5.28 × 10^−4^	23.1	0.985
**C2,50**	8.16 × 10^−5^	2.98 × 10^−5^	4.89 × 10^−4^	14.0	0.984
**C2,100**	6.33 × 10^−5^	5.01 × 10^−5^	8.37 × 10^−4^	95.1	0.997

It is clear that the capillary water absorption increased with the incorporation of FRCA. This property is basically determined by the porosity of concrete, which increases with the percentage of FRCA. Two reasons are given for this greater porosity in concrete made with FRCA: the FRCA’s greater porosity and the higher effective (w/c) ratio required to achieve the target slump value (Table 4).

A higher increase was noticed for the 100% replacement ratio, which is explained by the exponential growth of the connecting capillaries for this replacement ratio, to which Evangelista and de Brito (2010, [12]) refer. The family made without SP (C0) had an increase with respect to the reference concrete (RC0) of 10% for a replacement ratio of 50% and an increase of 45% for a replacement ratio of 100% in the 72 h test. The increase of the connecting capillaries was justified by the higher porosity of the FRCA and the higher (w/c) ratio. In fact, the mix made with 100% FRCA (C0,100) increases the (w/c)_ef_ ratio 7.8% relative to the reference mix (RC0).

**Figure 11:**
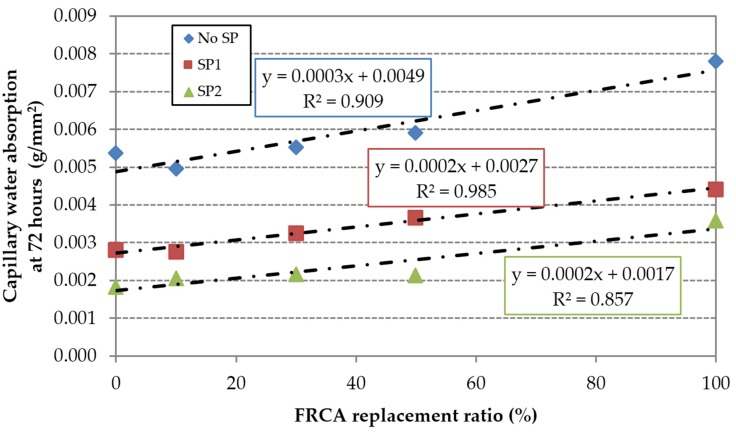
Capillary water absorption.

Similarly, the family made with SP1 (C1) increased from 31% to 58% for 50% and 100% of replacement ratios. The mix made with 100% FRCA (C1,100) increases the (w/c)_ef_ ratio by 16.3% relative to the reference mix (RC1). The family made with SP2 (C2) showed the greatest difference in the 72 h test, 15%–95% for 50% and 100% of replacement ratios: The mix made with 100% FRCA (C2,100) increases the (w/c)_ef_ ratio by 7.9% relative to the reference mix (RC2). A linear increase of the capillary with FRCA can be adjusted very well (Figure 11).

Concrete families made with SP showed the lowest values of sorptivity (Table 7). For each of the concrete families the sorptivity increased with the incorporation of FRCA except in the free superplasticizer family, in which a clear tendency was not appreciated by incorporating FRCA. Evangelista and de Brito [12] showed significant increases in the sorptivity with the incorporation of FRCA.

As in the water absorption by immersion, the replacement ratio of 10% showed a better performance in the concrete families without SP and with SP1. Due to their effectiveness in reducing the effective w/c ratio, the use of superplasticizers improves this property between 48% and 66% for RC1 and RC2 relative to RC0. The reference mixes (RC1 and RC2) needed a (w/c)_ef_ ratio 15.7% and 25.5% lower than RC0 (Table 4).

**Figure 12:**
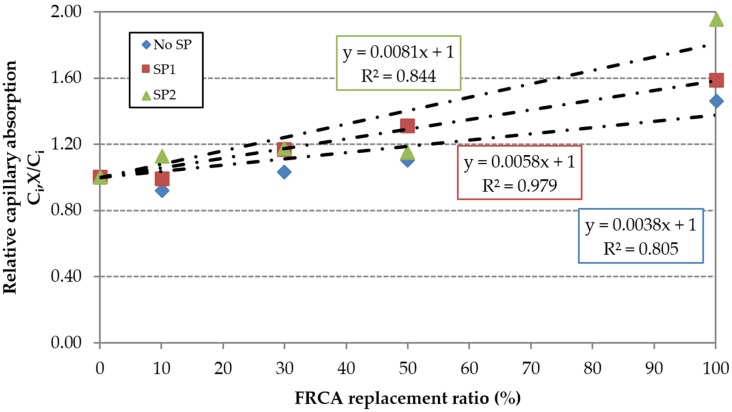
Influence of FRCA replacement ratio on the relative capillary water absorption.

The capillary water absorption values were slightly lower when 100% FRCA was used, *i.e.*, 43% and 54% for C1.100 and C2.100. In this case, the mixes C1,100 and C2,100 needed a (w/c)_ef_ ratio 9.1% and 25.5% lower than RC0. Mixes incorporating admixtures are more sensitive to the incorporation of FRCA due to the distribution changes caused by the SP, referred to by Mehta and Monteiro [47], which enhance the inter-connection of the capillaries. This is evidenced by the greater slope of the linear trend shown in Figure 12. The loss of effectiveness was greater in the high-performance SP2.

**Figure 13:**
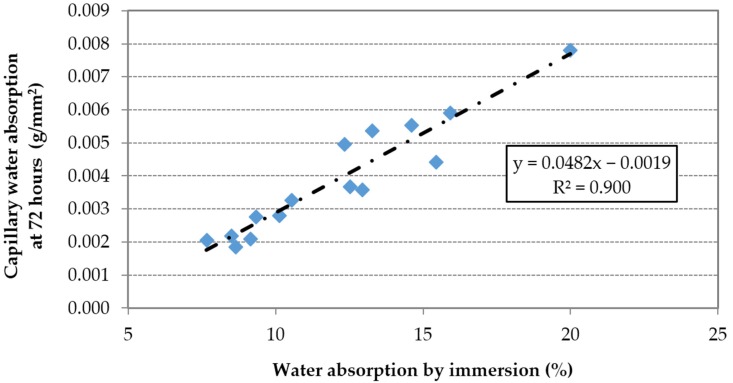
Capillary water absorption by immersion *vs.* water absorption by immersion.

A liner correlation between the capillary water absorption and the water absorption by immersion can be observed (Figure 13). The relationship between water absorption by immersion and effective w/c ratio was shown in Figure 6.

### 4.6. Carbonation Resistance

**Figure 14:**
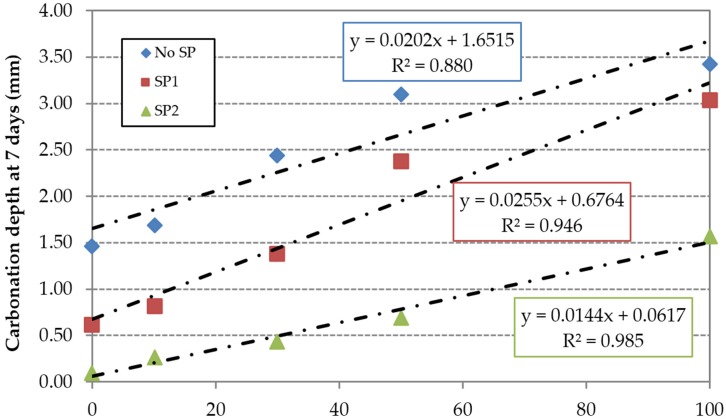
Carbonation depth at seven days.

**Figure 15:**
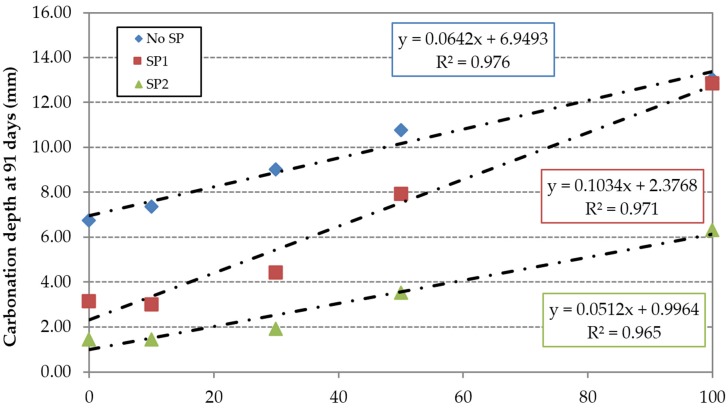
Carbonation depth at 91 days.

The carbonation depth for each of the concrete families *vs.* replacement ratio is shown in Figure 14 and Figure 15 at 7 and 91 days respectively. The carbonation depth increased with time. Concrete made with SP2 showed the lower carbonation depth values at any age. This can be related to the lower effective w/c ratio used in the mix (Table 3) resulting in a lower porosity and hence a lower permeability to gases [48]. As expected, the FRCA incorporation also increased the carbonation depth for all concrete families and test ages.

**Figure 16:**
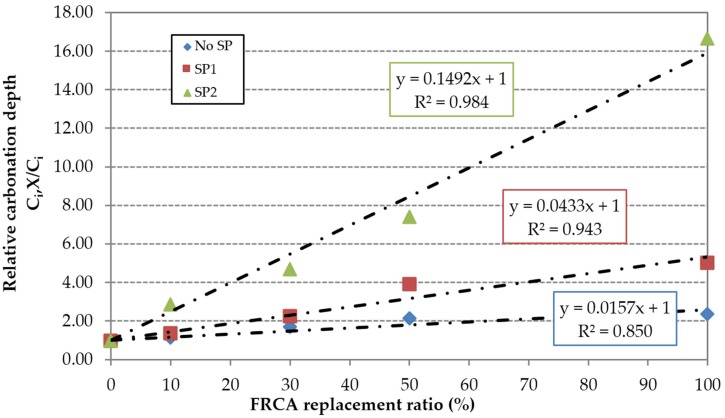
Influence of FRCA replacement ratio on the relative carbonation depth at seven days.

**Figure 17:**
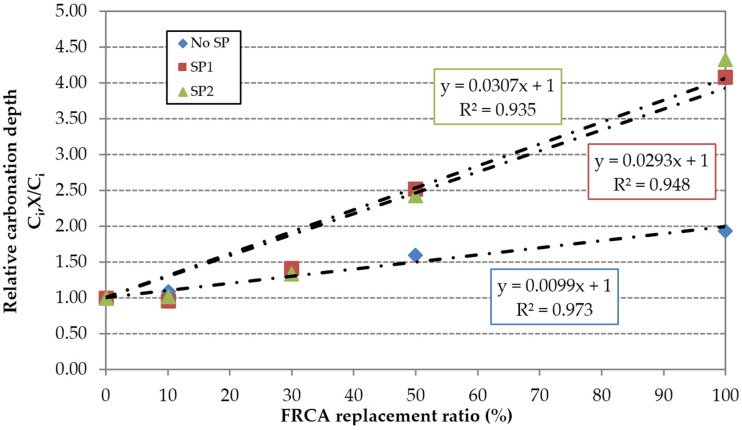
Influence of FRCA replacement ratio on the relative carbonation depth at 91 days.

In relative terms, the superplasticizers’ efficiency is compromised by the incorporation of FRCA, especially at early ages. This can be justified by the higher porosity of FRCA, which results in a greater contact surface between the aggregate particles and superplasticizers. The content used in the reference mixes RC1 and RC2 is insufficient to maintain the effectiveness of the superplasticizers. This is more evident in the SP2 family, as shown by the higher slope of the linear trend (Figure 16 and Figure 17); this is due to the low value of the carbonation depth of the RC2 mix. However, the use of high-performance superplasticizer compensated the negative effect of the FRCA for this property.

**Figure 18:**
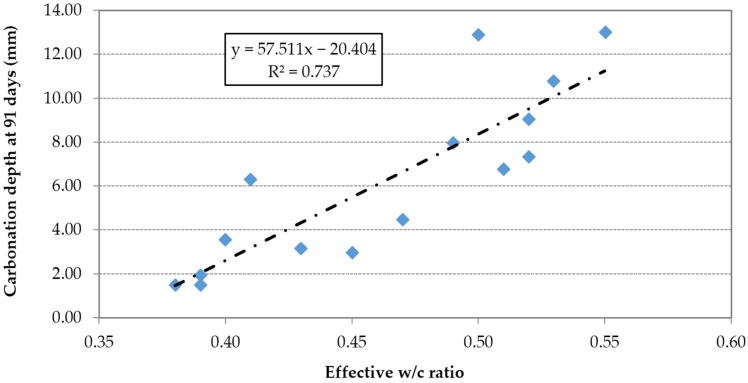
Carbonation depth *vs.* effective w/c ratio.

**Figure 19:**
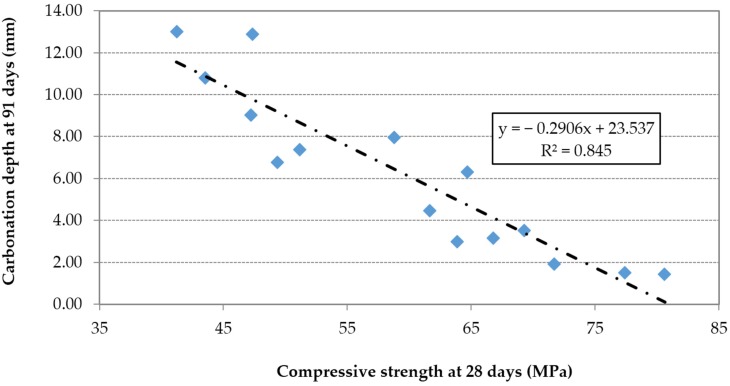
Carbonation depth *vs.* compressive strength.

The carbonation depth is proportional to the effective w/c ratio (Figure 18) and inversely proportional to the compressive strength (Figure 19).

Table 8 shows that concrete made with 100% FRCA and SP2 had a carbonation depth of 6.31 mm while the mix without superplasticizer and 100% FNA had 6.75 mm. This demonstrated that the increase of porosity caused by the use of FRCA is offset by the effect of high-performance SP in reducing the w/c ratio and increasing the compacity of concrete.

**Table 8:** 91-day carbonation depth of each of the concrete’s families and FRCA replacement ratios.

Family	C0			C1			C2		
FRCA (%)	Carbonation Depth (mm)	Δ_FRCA_ (%)	Δ_SP_ (%)	Carbonation Depth (mm)	Δ_FRCA_ (%)	Δ_SP_ (%)	Carbonation Depth (mm)	Δ_FRCA_ (%)	Δ_SP_ (%)
0	6.75	0.00	0.00	3.16	0.00	−53.24	1.46	0.00	−78.40
10	7.34	8.80	0.00	3.00	−4.95	−59.15	1.47	0.71	−80.00
30	9.03	33.80	0.00	4.44	40.59	−50.87	1.94	32.86	−78.55
50	10.78	59.72	0.00	7.94	151.49	−26.38	3.54	142.86	−67.15
100	13.03	93.06	0.00	12.88	307.92	−1.20	6.31	332.86	−51.56

### 4.7. Chloride Penetration Test

**Figure 20:**
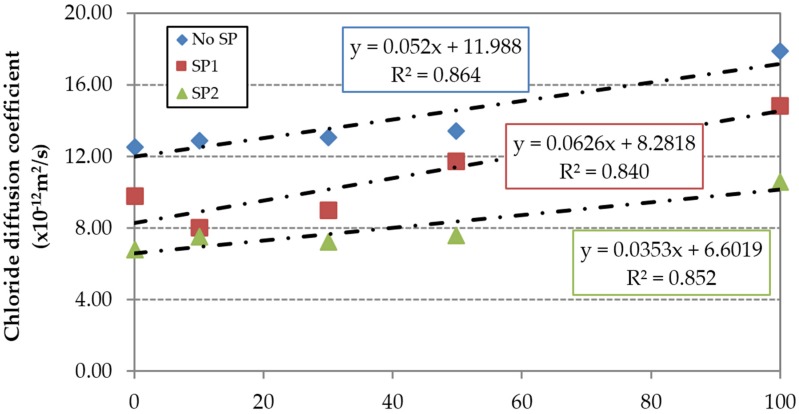
Chloride diffusion coefficient at 91 days.

The chloride penetration test results at 91 days are shown in Figure 20. There is a clear trend of increase of chloride diffusion coefficient by incorporating FRCA. Increases of 42%, 54% and 56% for concrete made without SP, with SP1 and with SP2, respectively, were obtained, which were related to the higher porosity of concrete made with FRCA.

**Figure 21:**
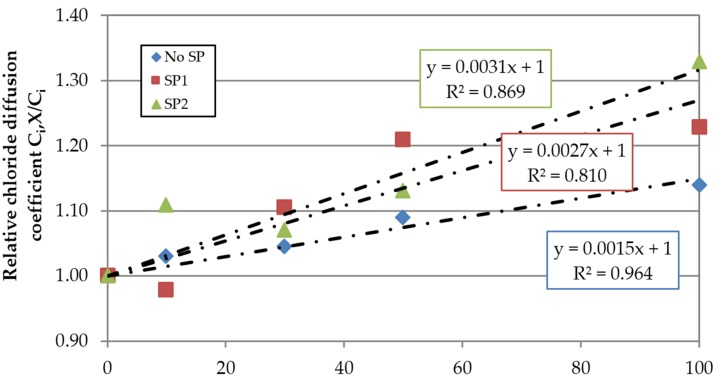
Influence of FRCA replacement ratio on the relative chloride diffusion coefficient at 91 days.

Maximum decreases of 38% and 46% were obtained for the concrete families made with SP1 and SP2 respectively, which was related to their lower effective w/c ratio. Figure 21 represents in relative terms the sensitivity of the superplasticizers to the FRCA incorporation, confirming the conclusions reached in the carbonation test. This effect is even clearer in the concrete made with SP2, as seen by the slope of the linear trend.

Table 9 shows the results for each of the concrete families and replacement ratios besides the values of Δ_FRCA_ and Δ_SP_. Concrete made with 100% FRCA and SP2 showed a chloride diffusion coefficient value of 10.60 × 10^−12^ m^2^/s, whereas the reference concrete RC0 showed a higher value of 12.57 × 10^−12^ m^2^/s. As in the carbonation depth, the loss of porosity achieved with the use of SP2 was greater than the increase resulting from the use of FRCA and the higher effective w/c ratio necessary in the mix to obtain similar slump.

**Table 9:** 91-day chloride diffusion coefficient of each of the concrete’s families and FRCA replacement ratios.

Family	C0			C1			C2		
FRCA (%)	D_nssm_ (10^−12^ m^2^/s)	Δ_FRCA_ (%)	Δ_SP_ (%)	D_nssm_ (10^−12^ m^2^/s)	Δ_FRCA_ (%)	Δ_SP_ (%)	D_nssm_ (10^−12^ m^2^/s)	Δ_FRCA_ (%)	Δ_SP_ (%)
0	12.57	0.00	0.00	9.79	0.00	−22.13	6.81	0.00	-45.80
10	12.91	2.74	0.00	8.01	−18.15	−37.96	7.49	10.00	-41.97
30	13.06	3.96	0.00	8.96	−8.40	−31.39	7.24	6.23	-44.62
50	13.38	6.50	0.00	11.69	19.47	−12.65	7.58	11.25	-43.39
100	17.90	42.43	0.00	14.85	51.73	−17.05	10.60	55.67	-40.77

**Figure 22:**
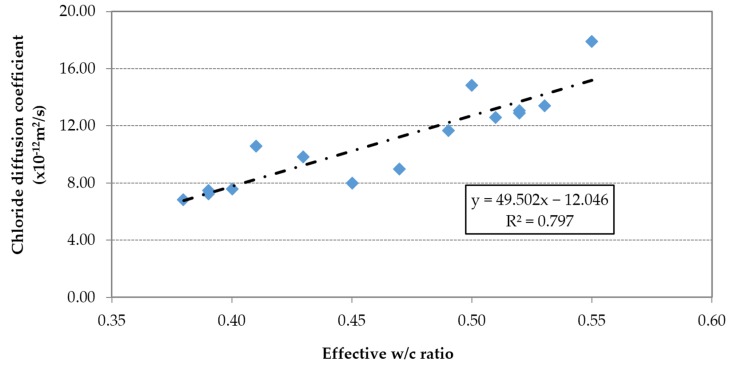
Chloride diffusion coefficient at 91 days *vs.* effective w/c ratio.

**Figure 23:**
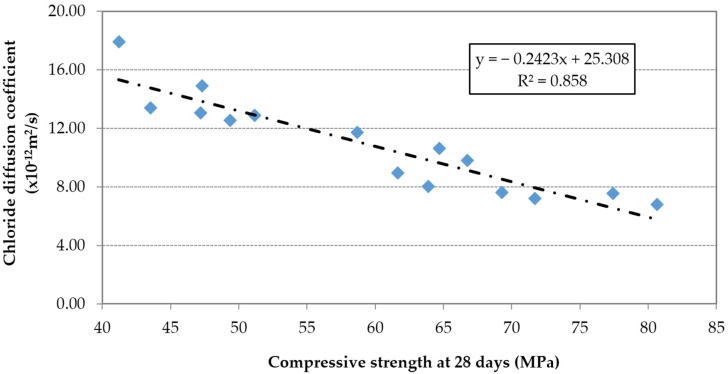
Chloride diffusion coefficient at 91 days *vs.* compressive strength.

The chloride diffusion coefficient was proportional to the effective w/c ratio (Figure 22) and inversely proportional to the compressive strength (Figure 23).

## 5. Conclusions

This paper demonstrates the feasibility of using FRCA in structural concrete production from a durability point of view. Three families of concrete were tested: concrete without SP, concrete made with a regular superplasticizer and concrete made with a high-performance superplasticizer. In each family, five volumetric replacement ratios of natural sand by FRCA were tested: 0%, 10%, 30%, 50% and 100%. The following conclusions can be drawn:

1. The incorporation of FRCA up to 100% had the following consequences on the concrete’s durability properties:

- The effective water/cement ratio increased up to 16%. The effect of FRCA was more pronounced in the family of concrete made with the regular superplasticizer;
- The water absorption by immersion increased linearly up to 52%. This property was similarly affected by the percentage of FRCA in the three families of concrete;
- The water absorption by capillary action in the 72 h test increased up to 45%. The effect of FRCA was more pronounced in the family of concrete made with the high-performance superplasticizer;
- The carbonation depth at 91 days increased up to 333%. The effect of FRCA was more pronounced in the family of concrete made with the high-performance superplasticizer;
- The chloride migration coefficient increased up to 33%. The effect of FRCA was more pronounced in the family of concrete made with the high-performance superplasticizer.

2. The addition of a regular superplasticizer had the following consequences on the concrete’s durability properties:

- The effective water/cement ratio decreased up to 16%. This percentage of variation decreased with the incorporation of FRCA;
- The water absorption by immersion decreased up to 28%. This percentage of variation was slightly reduced by the incorporation of FRCA;
- The water absorption by capillary in the 72 h test decreased up to 48%. This percentage of variation was reduced by the incorporation of FRCA;
- The carbonation depth at 91 days decreased up to 59%. This percentage of variation was strongly reduced by the incorporation of FRCA;
- The chloride migration coefficient decreased up to 38%. This percentage of variation was reduced by the incorporation of FRCA;
- The regular SP’s effectiveness was significantly jeopardized by the incorporation of FRCA.

3. The addition of a high-performance superplasticizer had the following consequences on the concrete’s durability properties:

- The effective water/cement ratio decreased up to 25%. The effect of the high-performance superplasticizer was not significantly affected by the incorporation of FRCA;
- The water absorption by immersion decreases up to 43%. This percentage of variation was not significantly affected by the incorporation of FRCA;
- The water absorption by capillary in the 72 h test decreased up to 66%. This percentage of variation was reduced by the incorporation of FRCA;
- The carbonation depth at 91 days decreased up to 80%. This percentage of variation was reduced by the incorporation of FRCA;
- The chloride migration coefficient decreased up to 46%. This percentage of variation was reduced by the incorporation of FRCA;
- The high-performance SP’s effectiveness was much more jeopardized than regular SP by the incorporation of FRCA, *i.e.*, the effect rises as the admixture’s quality increases.

4. In view of the results, the maximum FRCA replacement ratios that allow maintaining the RC’s performance for each of the durability properties are shown in Table 10.

**Table 10:** Maximum FRCA replacement ratios that can be offset in terms of durability properties by adding superplasticizers.

Durability Property	SP1	SP2
Water absorption by immersion	50% FRCA	100% FRCA
Water absorption by capillarity action	100% FRCA	100% FRCA
Carbonation resistance	30% FRCA	100% FRCA
Chloride penetration resistance	100% FRCA	100% FRCA

5. In conclusion, the simultaneous incorporation of FRCA and high-performance SP is a viable sustainable solution for structural concrete.

## Abbreviations

The following abbreviations are used in this manuscript:

SP: Superplasticizers
FRCA: Fine recycled concrete aggregate
RCA: Recycled concrete aggregates
CRCA: Coarse recycled concrete aggregates
FNA: Fine natural aggregate
